# Etiological Approach to Understanding Recanalization Failure in Intracranial Large Vessel Occlusion and Thrombectomy: Close to Embolism but Distant From Atherosclerosis

**DOI:** 10.3389/fneur.2020.598216

**Published:** 2021-01-18

**Authors:** Seong-Joon Lee, So Young Park, Ji Man Hong, Jin Wook Choi, Dong-Hun Kang, Yong-Won Kim, Yong-Sun Kim, Jeong-Ho Hong, Chang-Hyun Kim, Joonsang Yoo, Raul G. Nogueira, Yang-Ha Hwang, Sung-Il Sohn, Jin Soo Lee

**Affiliations:** ^1^Department of Neurology, Ajou University School of Medicine, Ajou University Medical Center, Suwon, South Korea; ^2^Department of Radiology, Ajou University School of Medicine, Ajou University Medical Center, Suwon, South Korea; ^3^Department of Neurosurgery, School of Medicine, Kyungpook National University, Daegu, South Korea; ^4^Department of Radiology, School of Medicine, Kyungpook National University, Daegu, South Korea; ^5^Department of Neurology, School of Medicine, Kyungpook National University, Daegu, South Korea; ^6^Department of Neurology, Keimyung University Dongsan Medical Center, Daegu, South Korea; ^7^Department of Neurosurgery, Keimyung University Dongsan Medical Center, Daegu, South Korea; ^8^Department of Neurology, National Health Insurance Service Ilsan Hospital, Goyang, South Korea; ^9^Department of Neurology, Marcus Stroke & Neuroscience Center, Grady Memorial Hospital, Emory University School of Medicine, Atlanta, GA, United States

**Keywords:** intracranial large vessel occlusion, recanalization failure, thrombectomy, middle cerebral artery, endovascular treatment

## Abstract

**Introduction:** In patients with intracranial large vessel occlusion (LVO) who undergo endovascular treatment (EVT), recanalization failure may be related to intracranial atherosclerotic stenosis (ICAS). We evaluated whether the risk factors of recanalization failure could possibly be a marker of ICAS among various types of LVO.

**Methods:** From a multicenter registry, patients with middle cerebral artery M1 segment occlusions who underwent thrombectomy within 24 h were included. Based on the on-procedure and post-procedure angiographic findings, patients were classified into embolic, ICAS-related, tandem occlusion, and recanalization failure groups. Recanalization failure was defined if the occluded vessel could not be recanalized by stent retrieval, contact aspiration, or local lytics treatment. Risk factors, imaging markers, and EVT methods were compared between groups.

**Results:** Among 326 patients, 214 were classified as embolism, 76 as ICAS, 16 as tandem, and 20 as recanalization failure. The group with recanalization failure showed higher scores on the National Institutes of Health Stroke Scale (NIHSS) (median, 16.0 vs. 14.5 vs. 14.0 vs. 17.0, *p* = 0.097), frequent atrial fibrillation (59.3 vs. 18.4 vs. 0 vs. 40.0% *p* < 0.001), and elevation in erythrocyte sedimentation rate (ESR) (14.5 ± 15.7 vs. 15.0 ± 14.1 vs. 21.2 ± 19.5 vs. 36.0 ± 32.9, *p* < 0.001) among the groups. The rate of computed tomography angiography-based truncal-type occlusion in recanalization failure group was not as high as that in the ICAS group (8.1 vs. 37.5 vs. 0 vs. 16.7%, *p* < 0.001). Balloon guide catheters (BGC) were less frequently utilized in the recanalization failure group as compared to their use in the other groups (72.0 vs. 72.4 vs. 62.5 vs. 30.0%, *p* = 0.001). In the multivariable analysis, initial higher NIHSS [odds ratio (OR), 1.11 95% confidence interval (CI), 1.01–1.22 *p* = 0.027], higher ESR (OR, 1.03 CI, 1.01–1.05 *p* = 0.006), and non-use of BGCs (OR, 3.41 CI, 1.14–10.17 *p* = 0.028) were associated with recanalization failure. In M1 occlusions, the predominant mechanism of recanalization failure was presumed to be embolic in 80% and due to ICAS in 20%.

**Conclusion:** The analysis of recanalization failures does not suggest an underlying predominant ICAS mechanism. Sufficient utilization of thrombectomy devices and procedures may improve the rates of recanalization.

## Introduction

Endovascular treatment (EVT) is an effective treatment for acute ischemic stroke due to large vessel occlusion (LVO) (1). Nevertheless, recanalization is not successful in all patients. Possible causes of recanalization failure include tandem occlusion, clot characteristics and its burden, or different occlusion pathomechanisms (atherosclerotic occlusions) apart from anatomical challenges that limit the initiation of mechanical thrombectomy itself (2–4).

More specifically, underlying intracranial atherosclerotic stenosis (ICAS) in LVO is associated with recanalization failure. It was reported that truncal-type occlusion (TTO), which is a marker of ICAS, was associated with stent retriever failure (5). Additionally, clot characteristics can be an important hurdle for recanalization. Despite recent studies regarding clots, it is difficult to evaluate which clot characteristics would result in recanalization failure because sufficient clot burden could not be retrieved. Instead, a hyperdense artery sign (HAS) on computed tomography (CT) may indirectly show some characteristic differences among clots in LVO.

In the current study, we first categorized acute stroke patients with large vessel occlusion into embolic, intracranial atherosclerosis-related, tandem occlusion, and recanalization failure groups. Thereafter, we aimed to identify the clinical features and imaging findings with clues for the identification of occlusion etiology in the recanalization failure group with special focus on ICAS etiology in LVO, thrombus characteristics, and EVT methods.

## Methods

### Study Population

Patients were retrospectively identified from the Acute Stroke due to Intracranial Atherosclerotic Occlusion and Neurointervention-Korean Retrospective (ASIAN KR) registry (6–8). Between January 2011 and May 2016, 720 patients who had undergone EVT for emergent large vessel occlusion were identified. From this registry, patients were included in the current study when (1) they had middle cerebral artery (MCA) M1 occlusion on baseline non-invasive angiography and underwent thrombectomy and (2) if the onset to puncture time was <24 h. Patients were excluded if the etiology of M1 was determined as dissection, Moyamoya disease, or vasculitis. After de-identification and blinding of clinical data, core laboratory imaging analysis was performed to ensure consistent grading and to eliminate bias. Initial large vessel occlusion location was identified on baseline CT or magnetic resonance angiography (S.J.L.). Infarct burden was measured using the Alberta Stroke Program Early CT Score (ASPECTS) (S.I.S.). Successful reperfusion was defined as a modified Treatment In Cerebral Ischemia grade of 2b−3 (9) (J.S.L. and Y.H.H.). The data collection protocol was approved by the Institutional Review Board of each hospital. This study was conducted in accordance with the ethical standards of the 1964 Declaration of Helsinki and its later amendments.

### Classification of Intracranial Large Vessel Occlusion Etiology

The etiology of MCA M1 occlusion was classified into four groups. The judgment of etiology was based on procedural angiography according to previous reports (Y.H.H. and J.S.L.) (6, 10). In brief, after confirmation of arterial occlusion, uncommon cerebral arterial diseases such as dissection, Moyamoya disease, and vasculitis were evaluated. If the occluded vessel was completely recanalized after primary thrombectomy such as stent retrieval, aspiration method, or local lytic methods but it was not involved in extracranial atherosclerosis, etiology was classified as embolic occlusion. If the occlusion was associated with extracranial atherosclerosis (i.e., tandem occlusion) and was completely recanalized, it was classified as tandem. Intracranial remnant stenosis of over 70%, moderate stenosis with tendency of re-occlusion, or flow impairment after successful recanalization through primary thrombectomy or additional lytic treatment was classified as ICAS. If the degree of recanalization could not be evaluated due to thrombectomy failure after stent retrieval, aspiration method, or lytic treatment, it was classified as recanalization failure. In this group, partial or complete reperfusion could be achieved by balloon angioplasty or permanent stent deployment, but their classification as recanalization failure was not altered. After the procedure, the occlusion etiology was further confirmed by evaluating repeat angiography during admission after EVT (J.Y.) to exclude possible dissection or vasospasm. Finally, the following groups were included in the analysis: (1) embolic, (2) ICAS, (3) tandem, and (4) recanalization failure groups.

### Imaging Analysis of Occlusion Types and Thrombus Characteristics

Previous studies have reported imaging markers to predict embolic occlusions and ICAS-related occlusions. A TTO sparing the major branches and their bifurcation sites beyond the occlusive segment was a predictive marker for ICAS-related LVO, while branching-site occlusion (BSO) involving the bifurcation site was a predictive marker for embolic origin of LVO (11, 12). For the current study, occlusion types were classified as either TTO or BSO based on baseline CT angiography by two individual interventional neurologists (S.J.L. and J.Y.) with consensus on discrepancy. For the MCA M1, if the bifurcation point and most proximal point of both M2 segments were visible, it was regarded as a TTO. Cases in which CT angiography images were not of sufficient quality to enable the differentiation between the two occlusion types were classified as inconclusive.

To analyze differences in thrombus burden (13), occlusion location (14), and evaluation of occlusion pathomechanism (15), HAS on non-contrast CT was evaluated (S.Y.P.). The location and distribution of HAS were evaluated by assessing involvement among four segments of the M1, and the total burden was scored on a scale of 0 to 4. The average Hounsfield units for the HAS were also evaluated.

### Statistical Analysis

Frequency and distribution were compared among embolic, atherosclerotic, and undetermined groups. Differences between the groups were analyzed using the χ^2^ test for categorical variables or analysis of variance. Direct comparison of frequency analyses and *post-hoc* Bonferroni test for continuous variables among groups were performed. A multiple logistic regression analysis was performed with clinically relevant variables for identification of risk factors for recanalization failure. A *p*-value of < 0.05 was considered significant. Statistical analysis was performed using the SPSS statistical package (version 22.0, Chicago, IL).

### Analysis of Recanalization Failure

In cases with recanalization failure, the patient data and imaging and procedural findings were carefully reviewed by experts to empirically conclude upon a presumed mechanism. A number of factors were considered into this analysis. Presence of atrial fibrillation favored cardioembolic etiology. Occlusion type on pre-procedure non-invasive angiographic images and upon stent retriever deployment was reviewed. TTO favored ICAS-related LVO, while BSO favored embolic etiology (11, 12). Presence of tandem occlusions proximal or distal to the main intracranial segment was evaluated. An atherosclerotic occlusion of the proximal ICA favored artery-to-artery embolisms, while distal ICA occlusions that completely recanalized, or tandem occlusions distal to main occlusions, were considered evidence for embolic etiology (16). Any thrombus migration distal to the initial occlusion site before or during EVT with no culprit stenosis was considered suggestive of embolic occlusions. The post-procedure non-invasive angiography performed usually on the next day was evaluated for delayed recanalization. If the delayed recanalization revealed a focal stenosis, it was considered evidence for ICAS, while complete recanalization was considered evidence for embolic occlusions (17).

## Results

### Baseline Demographics and Risk Factors

**Figure 1:**
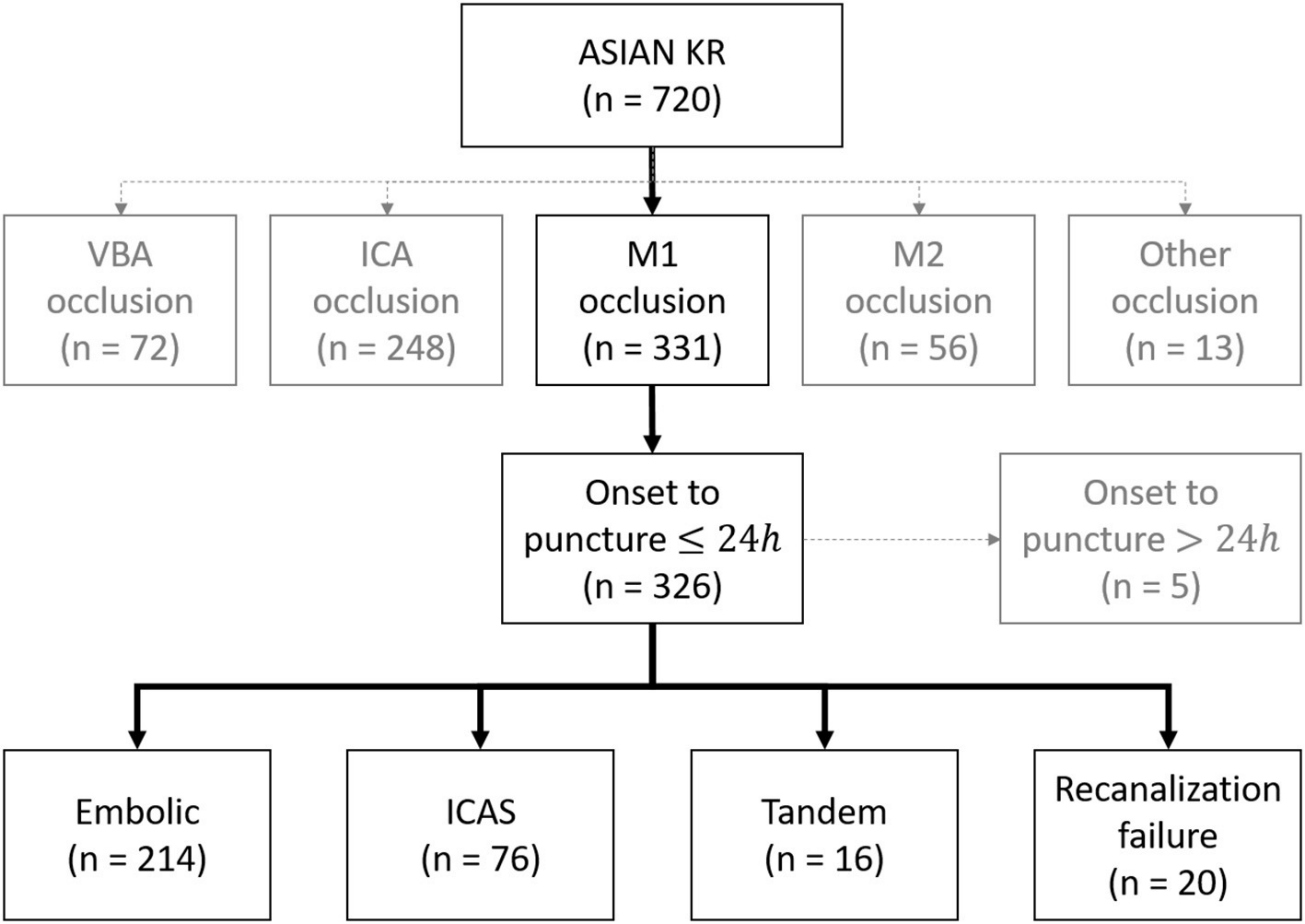
Classification of acute intracranial occlusion based on EVT. ASIAN KR, acute stroke due to intracranial atherosclerotic occlusion and neurointervention-korean retrospective; VBA, vertebrobasilar artery; ICA, internal carotid artery; ICAS, intracranial atherosclerotic stenosis.

A total of 326 patients were included in the analysis. Among them, 214 (65.6%) were classified as embolism, 76 (23.3%) as ICAS, 16 (4.9%) as tandem, and 20 (6.1%) as recanalization failure (Figure 1). Overall, embolic and failure groups shared similar clinical distributions, while tandem group and ICAS group shared some features (Table 1). Among the groups, atrial fibrillation was more frequent in embolic and failure groups (embolic, ICAS, tandem, and failure groups: 59.3, 18.4, 0, and 40.0%, respectively *p* < 0.001). National Institutes of Health Stroke Scale (NIHSS) scores tended to be higher in the failure group (15.9 ± 5.2, 14.5 ± 5.5, 14.6 ± 5.8, and 17.4 ± 7.8 *p* = 0.097), and the erythrocyte sedimentation rate (ESR) was the highest in the failure group (14.5 ± 15.7, 15.0 ± 14.1, 36.0 ± 32.9, 21.2 ± 19.5 *p* < 0.001). In contrast, male sex was more predominant in the ICAS and tandem groups (52.8 %, 67.1 %, 75.0 %, and 45.0 % *p* = 0.047). Smoking was more frequent in the ICAS and tandem groups (19.2 %, 35.5 %, 50.0 %, and 15.0 % *p* = 0.002), and low-density lipoprotein levels were also higher in the ICAS and tandem groups (99 ± 38, 118 ± 40, 107 ± 42, and 91 ± 34 mg/dl *p* = 0.001). Patients tended to be younger in the ICAS group (67 ± 13, 63 ± 14, 67 ± 8, and 67 ± 12 years *p* = 0.095).

**Table 1:** Baseline characteristics and risk factors of the four etiologic groups.

	**Embolism** **(*n* = 214)**	**ICAS** **(*n* = 76)**	**Tandem** **(*n* = 16)**	**Recanalization failure** **(*n* = 20)**	***P***
**Clinical characteristics**					
Age	67 ± 13	63 ± 14	67 ± 8	67 ± 12	0.095
Male sex	113 (52.8%)	51 (67.1%)	12 (75%)	9 (45%)	0.047
Initial NIHSS	16.0 [13.0–20.0]	14.5 [11.0–18.8]	14.0 [10.0–20.5]	17.0 [12.3–21.0]	0.097
Hypertension	128 (59.8%)	43 (56.6%)	10 (62.5%)	15 (75%)	0.514
Diabetes mellitus	45 (21.0%)	22 (28.9%)	8 (50%)	3 (15%)	0.031
Dyslipidemia	63 (29.4%)	18 (23.7%)	6 (37.5%)	3 (15%)	0.345
Smoking	41 (19.2%)	27 (35.5%)	8 (50%)	3 (15%)	0.002
Atrial fibrillation	127 (59.3%)	14 (18.4%)	0 (0%)	8 (40%)	<0.001
CAOD	25 (11.7%)	2 (2.6%)	2 (12.5%)	1 (5%)	0.105
Prior antiplatelet	62 (29.0%)	9 (11.8%)	5 (31.3%)	5 (25%)	0.027
Prior anticoagulant	33 (15.4%)	6 (7.9%)	1 (6.3%)	1 (5%)	0.194
Hemoglobin (g/dl)	13.3 ± 1.84	14.0 ± 1.9	13.2 ± 1.4	13.1 ± 1.8	0.024^*^
Hematocrit (%)	39.5 ± 5.3	41.2 ± 5.2	38.3 ± 4.1	38.5 ± 4.3	0.027^†^
Platelet (**× **10^3^)	214.6 ± 65.9	238.1 ± 66.7	282.8 ± 119.5	237.8 ± 95.3	0.001‡
WBC (**× **10^3^)	8.1 ± 2.8	9.7 ± 3.8	10.0 ± 3.4	9.2 ± 3.0	<0.001^*^
Initial glucose (mg/dl)	139.9 ± 52.1	149.8 ± 61.2	151.8 ± 63.0	136.1 ± 36.3	0.395
HbA1c	6.2 ± 1.3	6.3 ± 1.4	6.5 ± 1.5	6.5 ± 1.0	0.644
Total cholesterol (mg/dl)	166.7 ± 65.1	185.4 ± 43.2	179.6 ± 44.6	161.0 ± 41.7	0.091
TG (mg/dl)	105.3 ± 59.5	137.9 ± 158.6	224.4 ± 390.5	104.6 ± 74.0	0.002‡
HDL (mg/dl)	45.1 ± 11.0	45.0 ± 10.4	42.8 ± 12.6	44.1 ± 8.4	0.854
LDL (mg/dl)	99.4 ± 37.6	118.4 ± 39.6	107.3 ± 42.0	91.4 ± 34.0	0.001§
ESR (mm/h)	14.5 ± 15.7	15.0 ± 14.1	21.2 ± 19.5	36.0 ± 32.9	<0.001|
CRP (mg/dl)	0.7 ± 1.4	1.3 ± 4.4	1.5 ± 3.3	1.02 ± 2.2	0.227
**Imaging characteristics**					
ASPECTS	7.0 [5.0–9.0]	8.0 [6.0–9.0]	6.0 [4.0–7.0]	6.0 [6.0–8.8]	0.253
TTO on baseline angiography	14 (8.1%)	24 (37.5%)	0 (0%)	3 (16.7%)	<0.001
HAS burden	2 [2–3]	2 [2–4]	2 [2–2.75]	2 [2–3]	0.404
Hounsfield unit inHAS	60.7 ± 9.0	59.5 ± 8.7	60.2 ± 5.9	58.8 ± 6.7	0.668

The data are presented as the mean ± standard deviation, frequency (percentage), or median [interquartile range].

* Embolism vs. ICAS, p < 0.05, Bonferroni post-hoc test;

† embolism vs. ICAS, p = 0.07, Bonferroni post-hoc test; ‡embolism vs. tandem, p < 0.05, Bonferroni post-hoc test; §ICAS vs. embolism & recanalization failure, p < 0.05, Bonferroni post-hoc test; |recanalization failure vs. embolism & ICAS, p < 0.05, Bonferroni post-hoc test.

*ICAS, intracranial atherosclerotic stenosis; NIHSS, National Institutes of Health Stroke Scale; CAOD, coronary artery obstructive disease; WBC, white blood cell count; HbA1c, glycated hemoglobin; TG, triglyceride; HDL, high-density lipoprotein; LDL, low-density lipoprotein; ESR, erythrocyte sedimentation rate; CRP, C-reactive protein; ASPECTS, Alberta Stroke Program Early Computed Tomography Score; TTO, truncal-type occlusion; HAS, hyperdense artery sign*.

### Differences in Imaging Factors

**Figure 2:**
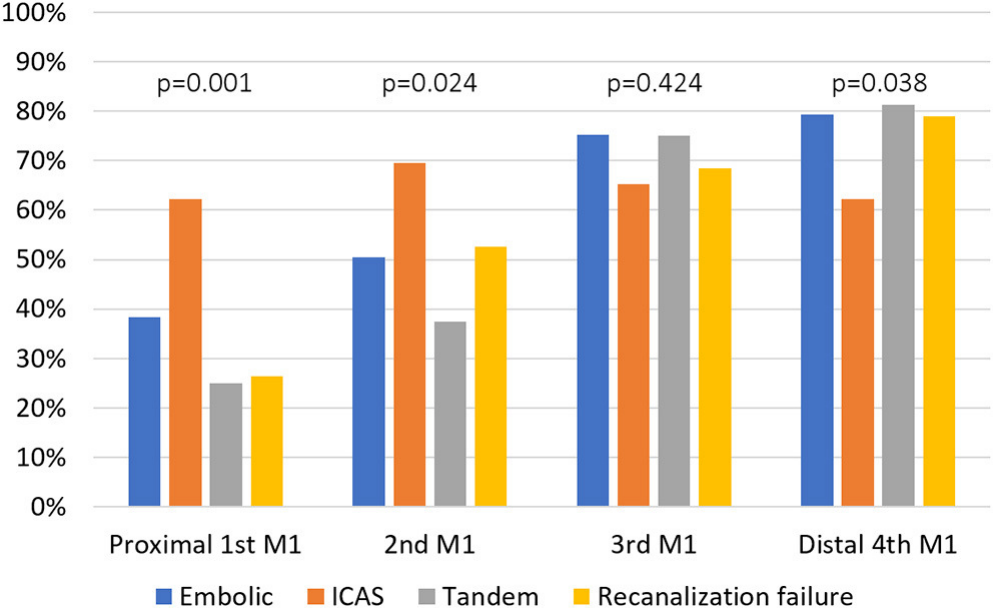
Comparison of hyperdense artery signs on the four respective segments of M1 segment between the groups. MCA, middle cerebral artery; ICAS, intracranial atherosclerotic stenosis.

Initial infarct burden as measured using the ASPECTS did not differ among the groups (*p* = 0.253) (Table 1). In terms of occlusion type, a TTO was more frequently observed in the ICAS group than in the other groups (8.1 vs. 37.5 vs. 0 vs. 16.7% *p* < 0.001). In the analysis of HAS distribution along the four segments of M1, HAS was most frequent in ICAS on the proximal first (38.4, 62.3, 25.0, and 26.3% *p* = 0.001) and the second segments (50.5, 69.6, 37.5, and 52.6% *p* = 0.024), while it was similarly frequent on the third segment among groups (75.3, 65.2, 75.0, and 68.4% *p* = 0.424), and it was less frequent in the ICAS group on the distal fourth segment (79.3, 62.3, 81.3, and 78.9% *p* = 0.038). The distribution of embolic, tandem, and recanalization failure groups did not differ (Figure 2). There were no differences in terms of total burden for the four points along the M1 segments (2 [2–3] vs. 2 [2–4] vs. 2 [2–2.75] vs. 2 [2–3] *p* = 0.404) or mean Hounsfield units of the HAS (60.7 ± 9.0 vs. 59.5 ± 8.7 vs. 60.2 ± 5.9 vs. 58.8 ± 6.7 *p* = 0.668), suggesting that there was no difference in thrombus characteristics.

### EVT Findings

Procedure time was the longest in the recanalization failure group (63 ± 37, 79 ± 40, 72 ± 36, and 110 ± 54 min *p* < 0.001). The use of a balloon guide catheter (BGC) was significantly less frequent in the failure group (72.0, 72.4, 62.5, and 30.0% *p* = 0.001). Final reperfusion success (modified Treatment in Cerebral Ischemia grade 2b−3) was less frequent in the failure group (81.3, 77.6, 75.0, and 10.0% *p* < 0.001), and 3-month good outcome (modified Rankin Scale 0–2) was less frequent in the failure group (60.1, 53.9, 62.5, and 15.0% *p* = 0.001) (Table 2).

**Table 2:** Treatment and outcomes according to the four etiologic groups.

	**Embolism** **(*n* = 214)**	**ICAS** **(*n* = 76)**	**Tandem** **(*n* = 16)**	**Recanalization failure** **(*n* = 20)**	***P***
Intravenous thrombolysis	118 (55.1%)	33 (43.4%)	8 (50.0%)	10 (50.0%)	0.371
Onset to puncture (min)	308 ± 208	408 ± 271	393 ± 315	347 ± 240	0.010^*^
Procedure time (min)	63 ± 37	79 ± 40	72 ± 36	110 ± 54	<0.001^†^
Onset to final angiography	371 ± 212	487 ± 253	464 ± 310	457 ± 253	0.001^*^
Use of balloon guide catheter	154 (72.0%)	55 (72.4%)	10 (62.5%)	6 (30.0%)	0.001
Stent retrieval, *n* (%)	115 (53.7%)	62 (81.6%)	3 (18.8%)	14 (70.0%)	<0.001
Direct aspiration, *n* (%)	148 (69.2%)	40 (52.6%)	10 (62.5%)	12 (60.0%)	0.075
Intracranial balloon, *n* (%)	0 (0%)	6 (7.9%)	0 (0%)	1 (5.0%)	<0.001
Intracranial stenting, *n* (%)	1 (0.5%)	7 (9.2%)	2 (12.5%)	1 (5.0%)	<0.001
First EVT method					<0.001
Penumbra 1^st^	94 (43.9%)	27 (35.5%)	3 (18.8%)	9 (45.0%)	
Penumbra MAX	38 (17.8%)	9 (11.8%)	1 (6.3%)	2 (10.0%)	
Solitaire	59 (27.6%)	30 (39.5%)	1 (6.3%)	5 (25.0%)	
Trevo	10 (4.7%)	7 (9.2%)	0 (0.0%)	0 (0.0%)	
Lytic infusion	1 (0.5%)	0 (0.0%)	0 (0.0%)	0 (0.0%)	
Angioplasty	5 (2.3%)	3 (3.9%)	7 (43.8%)	2 (10.0%)	
Remote aspiration	7 (3.3%)	0 (0.0%)	4 (25.0%)	1 (5.0%)	
Others	0 (0.0%)	0 (0.0%)	0 (0.0%)	1 (5.0%)	
Number of techniques, median [IQR]	1 [1–2]	2 [1.25–2.75]	2 [1–2]	2 [1–3]	<0.001
Successful recanalization (AOL 2–3)	207 (96.7%)	53 (69.7%)	11 (78.6%)	3 (15%)	<0.001
Successful reperfusion (mTICI 2b−3)	174 (81.3%)	59 (77.6%)	12 (75.0%)	2 (10.0%)	<0.001
PH2 or SAH 3–4	11 (5.1%)	5 (6.6%)	1 (6.3%)	5 (25%)	0.009
Favorable outcomes	128 (60.1%)	41 (53.9%)	10 (62.5%)	3 (15.0%)	0.001

The data are presented as the mean ± standard deviation, frequency (percentage), or median [interquartile range].

* Embolism vs. ICAS, p < 0.05, Bonferroni post-hoc test; †Recanalization failure vs. embolism & ICAS & tandem, ICAS vs. embolism, p < 0.05, Bonferroni post-hoc test.

*ICAS, intracranial atherosclerotic stenosis; IQR, interquartile range; AOL, arterial occlusive lesion score; mTICI, modified Treatment in Cerebral Ischemia score; PH, parenchymal hematoma; SAH, subarachnoid hemorrhage*.

### Factors Associated With Recanalization Failure and Presumed Pathomechanism

In the multivariable analysis of factors associated with recanalization failure, factors associated with occlusion etiology such as atrial fibrillation, proximal M1 involvement, or TTO patterns did not predict recanalization failure. Instead, significant factors were NIHSS [odds ratio (OR): 1.11, 95% confidence interval (CI): (1.01–1.22), *p* = 0.027], ESR [OR: 1.03, 95% CI: (1.01–1.05), *p* = 0.006], and non-use of BGC [OR: 3.41, 95% CI: (1.14–10.17), *p* = 0.028], suggesting the influence of systemic inflammation and procedural factors (Table 3). Upon careful review of each recanalization failure case, 16/20 (80.0%) were presumed to be embolic in origin, while 4/20 (20.0%) were presumed to be ICAS-related occlusions (Table 4). In cases of follow-up angiography, delayed recanalization was rare.

**Table 3:** Predictors for recanalization failure.

**Variables**	**Odds ratio (95% CI)**	***P***
Age	–	
Atrial fibrillation	–	
NIHSS	1.11 (1.01–1.22)	0.027
ESR, per 1 mm/h increase	1.03 (1.01–1.05)	0.006
Truncal-type occlusion on baseline angiography	–	
HAS burden	–	
HAS M1 first segment involvement	–	
Non-use of balloon guide catheter	3.41 (1.14–10.17)	0.028

*NIHSS, National Institutes of Health Stroke Scale; ESR, erythrocyte sedimentation rate; HAS, hyperdense artery sign*.

**Table 4:** Description of the 20 recanalization failure patients according to etiologic classification and expert opinion on presumed etiology.

**Number**	**Age**	**Sex**	**NIHSS**	**ASPECTS**	**A-fib**	**Baseline** **occlusion pattern**	**Procedure** **findings**	**Post-procedure angio**	**Presumed mechanism**
1	69	F	31	2	Y	TTO	Tandem distal ICA and M1 without ECAS	NA	Embolic (CE)
2	83	M	14	4	Y	BSO	Thrombus migration Culprit ulcerative ICA stenosis	Recanalization	Embolic (AA)
3	57	M	12	9	N	BSO	TTO on stent deployment, reocclusion	Occlusion	ICAS
4	70	M	23	6	Y	BSO	Visible thrombus	Occlusion	Embolic (CE)
5	77	F	19	6	N	BSO	BSO on stent deployment, procedural A1 embolization	NA	Embolic (Cryp)
6	64	F	19	3	Y	BSO	Ulcerated carotid plaque	Occlusion	Embolic (AA)
7	65	M	21	2	N	BSO	Persistent occlusion	NA	Embolic (Cryp)
8	82	F	18	6	N	TTO	Persistent occlusion	NA	ICAS
9	70	M	14	9	Y	BSO	BSO on stent deployment	Occlusion	Embolic (CE)
10	64	F	4	10	N	NA	BSO on stent deployment	Partial recanalization	ICAS
11	43	F	11	8	N	BSO	BSO on stent deployment, visible thrombus	NA	Embolic (Cryp)
12	73	M	11	6	N	BSO	Reocclusion, ICAS or dissection	Occlusion	ICAS
13	63	F	21	6	N	BSO	Thrombus migration	NA	Embolic (Cryp)
14	75	F	5	8	Y	NA	BSO on stent deployment	Occlusion	Embolic (CE)
15	65	F	13	6	Y	BSO	Thrombus migration	Occlusion	Embolic (CE)
16	34	M	19	7	N	BSO	Persistent occlusion	NA	Embolic (Cryp)
17	75	F	16	6	N	BSO	BSO on stent deployment, thrombus migration	NA	Embolic (Cryp)
18	71	M	24	7	N	BSO	BSO on stent deployment	Occlusion	Embolic (Cryp)
19	76	F	37	10	Y	TTO	Tandem distal ICA and M1 occlusion without ECAS	Occlusion	Embolic (CE)
20	73	M	16	9	N	BSO	Tandem distal ICA and MCA occlusion	Occlusion	Embolic (AA)

*NIHSS, National Institutes of Health Stroke Scale; ASPECTS, Alberta Stroke Program Early Computed Tomography Score; A-fib, atrial fibrillation; TTO, truncal-type occlusion; ICA, internal carotid artery; ECAS, extracranial atherosclerotic stenosis; CE, cardioembolic; BSO, branching-site occlusion; AA, artery-to-artery; ICAS, intracranial atherosclerotic stenosis; Cryp, cryptogenic source*.

## Discussion

In the current study, the major finding was that recanalization failure in mechanical thrombectomy for LVO was not associated with underlying ICAS based on angiographic evaluation or thrombus characteristics based on CT HAS. Instead, initial high severity score, initial high ESR levels, and non-use of BGC were proven to be associated with recanalization failure.

How recanalization failure is defined is an important issue that needs to be addressed because its definition has been varied among previous studies. First, recanalization should be distinguished from reperfusion, although both terms represent the same situations in most cases (18). For intracranial LVO, complete reperfusion does not always follow complete recanalization, especially when small branches are occluded. In contrast, partial recanalization can be followed by complete reperfusion in the underlying ICAS cases among LVOs. Even though reocclusion of partial recanalization repeatedly occurs during mechanical thrombectomy, complete reperfusion can be achieved after the remnant stenosis site is stabilized by intra-arterial antiplatelet treatments (19, 20). During the neurointervention, an unfavorable response to intracranial EVT may fall into the three categories as follows (3–5, 21): (1) access failure due to vascular tortuosity or external carotid artery stenosis (2) thrombectomy refractoriness possibly due to fibrin clot and (3) partial recanalization or reocclusion on stent retrieval or contract aspiration. For the current study, partial recanalization was classified into the ICAS group. In addition, intracranial balloon angioplasty or stent insertion upon recanalization failure without partial recanalization was classified into the failure group.

Another finding of our study is that recanalization failure in mechanical thrombectomy for LVO was not predominated by underlying ICAS. In the early generations of mechanical thrombectomy, the frequency of underlying atherosclerotic occlusions and its effect on recanalization failure were not completely understood. However, with primary stent retrieval, temporary bypass is usually seen in most patients (19). Analysis of large cohort data further showed that with appropriate rescue therapy such as intra-arterial tirofiban, angioplasty, or stent placement, the rates of reperfusion, or outcomes, may be similar when compared to those of embolic occlusions (6, 22). However, in cases where even temporary bypass is not achieved, how much does ICAS account for recanalization failure remains to be understood. The results of our study show that there is no predominance of atherosclerosis. The risk factors of recanalization failure were closer to embolic occlusion rather than ICAS-related occlusion. The frequency of atrial fibrillation, a major embolic source, was higher in both embolic and failure groups but less in the ICAS group. Well-known risk factors of atherosclerosis were not predominant in the failure group. In terms of imaging findings, the TTO pattern, a marker for ICAS in LVO, was less frequent in the failure group. Upon the HAS localization along the MCA M1 segment, it was found to be proximally located in the ICAS group, whereas it was more distally located in the embolic, tandem, and failure groups. Additionally, the assessment of presumed mechanism, which was evaluated *post-hoc* by expert opinion, showed that embolic occlusion might account for 80% of recanalization failure groups, while ICAS LVO accounts for 20%. These findings do not support the notion that recanalization failure may be primarily associated with ICAS LVO.

In contrast, non-use of BGC was a significant predictor of recanalization failure. Along with contemporary mechanical thrombectomy devices such as stent retrieval and large-bore aspiration catheters, our results provide further evidence that BGCs significantly contribute to improvements in recanalization (23, 24). These consistent results highlight the importance of improvements in devices and thrombectomy techniques in achieving higher rates of reperfusion. In a study wherein stent-retriever thrombectomy was performed, no reperfusion was achieved in 10.6% cases (25). Among these failures, most were due to technical difficulties, stent-retriever failure, and inability to reach target occlusion. We agree that technical development and scientific effort should focus on thrombectomy efficacy as well as tools and alternative access routes to improve cervical and intracranial access. In addition to ICAS cases, when encountered with an intractable occlusion, the rational method would be to increase efforts for thrombus removal, rather than angioplasty of the vessel wall, until there is evidence otherwise.

Further, in our study, thrombus characteristics were not associated with a specific occlusion etiology such as embolism or ICAS, which is in agreement with a previous meta-analysis (26). It is generally considered that a red blood cell-dominant clot shows higher attenuation and is more responsive to mechanical thrombectomy, while an intractable fibrin clot is likely to have more hypoattenuation and absence of HAS (26). However, large published cohorts evaluating thrombus attenuation have not shown increased rates of reperfusion (27, 28). Research is needed to establish if thrombus composition affects reperfusion outcomes and if CT-based attenuation evaluation is the most sufficient way to detect thrombus composition.

Elevated ESR was associated with recanalization failure in this study. There is evidence that various inflammation-based scores are associated with outcomes after mechanical thrombectomy (29). Furthermore, inflammation is associated with thrombus formation. There is evidence that neutrophil extracellular traps, a form of neutrophil-mediated immunity, contribute to ischemic stroke thrombi (30). Clots associated with large artery atherosclerosis showed significantly higher interleukin-1β expression tumor necrosis factor-α and matrix metalloproteinase-9 expression was significantly higher in clots with a negative susceptibility vessel sign than in those with a positive susceptibility vessel sign (31). We can extrapolate from our results that an inflammatory mechanism may play a role in thrombi with recanalization failure. Targeting such mechanisms may have potential.

This study has some limitations. First, as the classification of pathomechanism is not based on pathologic confirmation, it may be subject to errors. Nevertheless, the ICAS definition of the current study has been utilized numerous times in similar studies (6, 10, 15). For recanalization failure, we focused on recanalization failure without treatment methods that can potentially alter the morphology of the arterial structure, as such would result in alteration of the classification of pathomechanism. We clearly define this in the *METHODS* section. Second, while we performed analysis of thrombus characteristics by evaluation of HAS and vessel attenuation, the CT analysis used was 5-mm thick and may introduce a certain margin of error due to partial volume effect (32). The best imaging methods to describe thrombus characteristics and if thrombus characteristics indeed affect the response to mechanical thrombectomy need further study. Third, while we used preprocedure CT-based occlusion-type analysis in this study, the sensitivity for detection of fixed focal stenosis in the MCA vascular bed is modest, while the specificity is high (12). Application of TTO on a case-by-case basis may be liable to errors. Occlusion-type analysis after stent retriever deployment could not be applied to our study due to the heterogeneous reperfusion modality used and was used only for clues of occlusion etiology in the recanalization failure group. However, in the recanalization failure group, the rates of CTA-based TTO were much lower than those of the ICAS group and approached that of the embolic group. Fourth, our results regarding thrombus burden may be biased by our selection criteria, which included only patients with M1 occlusions. In terms of thrombus burden, poor outcomes (33) have been reported with increasing thrombus burden, while other studies have shown no association between clot burden score and outcomes or reperfusion (34). It is generally understood that thrombectomy is effective in achieving successful recanalization and good clinical outcomes throughout the entire range of clot burden score values. Further studies are needed to address these issues.

In conclusion, through a stepwise approach to evaluate occlusion etiology and thrombus imaging analysis in acute ischemic stroke patients with MCA M1 occlusion, we could identify that recanalization failure was neither predominantly due to differences in target vessel pathology such as ICAS nor was it associated with differences in thrombus characteristics. Improvements in mechanical thrombectomy devices and procedures, such as BGCs, and addressing the role of inflammation in thrombus formation may be potential targets to improve recanalization.

## Data Availability Statement

The raw data supporting the conclusions of this article will be made available by the authors, without undue reservation.

## Ethics Statement

The studies involving human participants were reviewed and approved by Ajou university hospital IRB. Written informed consent for participation was not required for this study in accordance with the national legislation and the institutional requirements.

## Author Contributions

S-JL, SP, and JL contributed to the conception and design of the study, acquisition and analysis of data, and preparation of the manuscript. Y-HH, S-IS, JH, JC, D-HK, Y-WK, Y-SK, J-HH, JY, and C-HK contributed to acquisition and analysis of data and reviewed the manuscript for critical intellectual content. RN reviewed the manuscript for critical intellectual content. All authors contributed to the article and approved the submitted version.

## Conflict of Interest

The authors declare that the research was conducted in the absence of any commercial or financial relationships that could be construed as a potential conflict of interest.

## Abbreviations

ASPECTS: alberta stroke program early CT score
BGC: balloon guide catheters
BSO: branching-site occlusion
CT: computed tomography
ESR: erythrocyte sedimentation rate
EVT: endovascular treatment
HAS: hyperdense artery sign
ICAS: intracranial atherosclerotic stenosis
LVO: large vessel occlusion
MCA: middle cerebral artery
NIHSS: National Institutes of Health Stroke Scale
TTO: truncal-type occlusion.
